# Recent advances in understanding rhinovirus immunity

**DOI:** 10.12688/f1000research.15337.1

**Published:** 2018-09-24

**Authors:** Spyridon Makris, Sebastian Johnston

**Affiliations:** 1 National Heart and Lung Institute, Medical Research Council and Asthma UK Centre in Allergic Mechanisms of Asthma, Imperial College London, London, UK

**Keywords:** Rhinoviruses, Asthma

## Abstract

Rhinoviruses are the most common cause of upper respiratory tract infections. However, they can induce exacerbations of chronic obstructive pulmonary disease and asthma, bronchiolitis in infants, and significant lower respiratory tract infections in children, the immunosuppressed, and the elderly. The large number of rhinovirus strains (currently about 160) and their antigenic diversity are significant obstacles in vaccine development. The phenotype of immune responses induced during rhinovirus infection can affect disease severity. Recognition of rhinovirus and a balance of innate responses are important factors in rhinovirus-induced morbidity. Immune responses to rhinovirus infections in healthy individuals are typically of the T helper type 1 (Th1) phenotype. However, rhinovirus-driven asthma exacerbations are additionally characterised by an amplified Th2 immune response and airway neutrophilia. This commentary focuses on recent advances in understanding immunity toward rhinovirus infection and how innate and adaptive immune responses drive rhinovirus-induced asthma exacerbations.

## Introduction

Rhinoviruses (RVs) are the main cause of common colds and are responsible for the majority of acute exacerbations of chronic obstructive pulmonary disease (COPD) and asthma ^1, 2^. Despite being discovered over 50 years ago, RVs have no effective anti-viral treatment or vaccine. Recently, the number of RV strains identified increased to about 160 RV strains/subtypes. RVs are divided into three species—RV-A, -B, and -C—on the basis of genetic classification. Species A and B are also classified on the basis of the cell surface receptor used for cellular entry. The “major group” of RVs, which includes about 90% of the 100 serotyped strains, uses intercellular adhesion molecule-1 (ICAM-1), whereas the “minor group” (~10% of the 100 serotyped strains) gains cell entry via the low-density lipoprotein receptor (LDLR) ^3, 4^. RV-C (~60 non-serotyped strains) uses cadherin-related family 3 (CDHR3) for binding and replication ^5^. The high number and variability of RV serotypes make vaccine design extremely challenging.

Nasal and bronchial epithelial cells are the first targets of RVs and are responsible for initiating anti-viral responses ^6^. Viral recognition occurs via various pattern recognition receptors (PRRs), including RIG-I-like receptors (RLRs) and Toll-like receptors (TLRs), which induce the production of inflammatory mediators and interferons (IFNs). The role of IFNs is important for the control of RV infection, as they induce anti-viral IFN-stimulated genes and inflammatory mediators which limit viral replication (the role of inflammatory mediators during RV infection and asthma has recently been reviewed ^6, 7^). This inflammatory “cascade” induces the recruitment and activation of innate cells which can affect the phenotype of adaptive responses. RVs enhance interleukin-25 (IL-25), IL-33, and thymic stromal lymphopoietin (TSLP) which drive type 2 immune responses ^8– 11^. Increasing evidence suggests that the recognition of RVs, the type of innate cells, and the production of inflammatory mediators can drive the phenotype of immune responses. This commentary focuses on very recent advances made in understanding how innate/adaptive immune responses drive RV-induced asthma exacerbations.

## Rhinovirus infection

RVs are positive-sense, single-stranded RNA (ssRNA) viruses belonging to the *Picornaviridae* family and *Enterovirus* genus. RVs are the most common cause of upper respiratory tract infection and are linked to exacerbations of COPD and asthma. Furthermore, they are the cause of bronchiolitis in infants and lower respiratory tract infections in children, the immunosuppressed, and the elderly ^4^. About 160 serotypes/strains of RV have been identified and are classified into three species: RV-A (74 serotypes), RV-B (25 serotypes), and RV-C (~60 strains) ^12– 14^. RV-C species were the last to be identified and are responsible for more severe illness, wheezing, and asthma exacerbations ^3, 4^. The RV viral capsid sequence variation causes the antigenic diversity between strains. The viral capsid is composed of VP1, VP2, VP3, and VP4 proteins. VP1 and VP3 are important for viral attachment to cell surface receptors in ciliated nasal and bronchial epithelial cells ^15, 16^.

Large-scale birth cohort studies identify a relationship between wheezing RV infections in early life and development of asthma later in high-risk children ^17^. RV-C infections are associated with severe infection in children, and a *CDHR3* gene mutation can mediate enhanced RV-C entry to host cells ^5^. Recent clinical evidence, from two birth cohorts, suggests that a *CDHR3* mutation is an asthma risk allele associated with enhanced RV-C illness ^18^. Targeting the interaction between RV and respective adhesion receptors can provide a therapeutic avenue. The precise mechanisms by which RVs induce asthma exacerbation are unclear. Impaired anti-viral immunity can influence the onset of infection in bronchial epithelial cells (BECs) derived from patients with asthma ^19– 21^. Furthermore, evidence suggests that multiple immunophenotypes of immune responses to RV exist in childhood that enhance asthma development ^22^. Improved understanding of how RV infection drives the phenotype of infection can help distinguish potential therapeutic and vaccine targets against RV-induced asthma exacerbations.

## Innate recognition of rhinovirus

Following RV infection of airway epithelial cells, a series of cellular PRRs can recognise viral antigens. TLR2 on the cell surface recognises viral capsid proteins ^23^. Within the endosome, TLR3 and TLR7/8 recognise viral double-stranded RNA (dsRNA) or ssRNA, respectively ^24, 25^. The recognition of RV by TLR3 in BECs also induces the expression of RIG-I and MDA5, which can recognise ssRNA and dsRNA, respectively ^23^. Recognition of RV by PRRs causes the secretion of inflammatory cytokines, including IL-6, tumour necrosis factor, IL-12, IL-15, and type I and type III IFNs. This also drives the secretion of chemokines such as CXCL10 (IP-10) and CXCL8/IL-8 which drive the recruitment of T cells—monocytes, natural killer cells, and dendritic cells (DCs) are also recruited—and neutrophils, respectively ^26^. Further to IFNs and inflammatory cytokines, epithelial cells are also a source of IL-33, IL-25, and TSLP, all of which can drive T helper type 2 (Th2) cell responses during RV infection (RV-induced cytokines have been extensively reviewed ^6, 7, 27^). Evidence suggests that anti-viral defence within bronchial epithelium requires co-ordinated recognition of RV ^28^.

The events that occur during innate recognition may help understand how asthma and COPD exacerbations develop after RV infection. Primary BECs from patients with asthma have a deficiency in TLR3 and MDA5 signalling, which reduces the production of inflammatory mediators after RV infection ^25, 26^. TLR3 expression in asthma is not impaired, and blocking the receptor in mice does not have a significant effect in reducing viral replication ^26, 29^. In contrast, blockade of TLR3, using CNT03157, in healthy volunteers reduces the production of inflammatory mediators and cellular recruitment after RV16 infection (thus reducing cold symptoms); however, it had no effect in asthma ^30^.

TLR7/8 are expressed in a number of lung cells, including epithelial cells, macrophages, and DC subsets. Plasmacytoid DCs (pDCs) respond rapidly to TLR7 ligation and induce type I IFN production because of a constitutive expression of IFN regulatory factor-7 (IRF-7) ^31, 32^. The mechanisms behind why the production of IFNs in asthma is impaired during RV infection are poorly understood. RV infection of mice with impaired TLR7 signalling showed a reduced production of IFNs, eosinophilia, and airway hyper-reactivity ^24^. The effects were reversed by adoptive transfer of TLR7-competent pDCs or exogenous IFN. Furthermore, Th2 cytokine induction after RV infection is a negative regulator of TLR7- or TLR3-induced IFNs ^24^. The induction of IL-4 and IL-13 inhibits the expression of TLR3 and IRF-3 and impairs the immune responses of epithelial cells against RVs ^33^. These observations support evidence that patients with severe asthma have reduced TLR7 expression and IFN production in the bronchoalveolar lavage ^34, 35^. Overall, complex innate recognition is involved during RV infection with a co-ordinated recognition, and an early robust IFN response and balance in the inflammatory mediators are essential for viral clearing and minimising RV-induced morbidity ^21, 33^.

## Interplay between innate and adaptive responses drives rhinovirus-induced exacerbations

Recognition of RV antigens by T cells initiates cytotoxic T-cell responses and activates T helper cells that drive humoral responses. Upon infection, epithelial cells, macrophages, and recruited neutrophils secrete CXCL10 (IP-10), which increases the chemotaxis of T cells ^4, 36^. The type of T-cell response toward RV is one of the factors driving asthma exacerbations ^37^. In healthy individuals, the primary immune responses to RV infection are T helper type 1 (Th1) and are characterised by a release of IFN-γ ^38^. Th2 immune responses are characterised by increased production of IL-4, IL-5, and IL-13 and have been associated with RV infection in asthma ^6, 10, 37, 38^. Cytokines inducing Th2 responses provide a potential therapeutic target. For example, dupilumab (IL-4 and IL-13 signalling inhibitor) significantly reduces the rates of severe asthma exacerbations and improves lung function in patients ^39^. Recent studies also indicate an increasingly important role for innate lymphoid cells (ILCs) in driving RV infections ^10, 40– 42^. Specifically, ILC2s are elevated in patients with asthma and are potent producers of type 2 cytokines, which can drive adaptive immune responses ^6, 27, 42, 43^.

Immune responses to respiratory infections in healthy individuals are typically characterised by a Th1 immune response. However, RV-driven asthma exacerbations are additionally characterised by an amplified Th2 immune response and airway neutrophilia ^44^. Neutrophil degranulation and elastase release in the airways are believed to contribute to obstruction in the lower airways in RV-induced asthma exacerbations ^45^. The mechanisms behind how RV drives Th2 immune responses during asthma exacerbations were recently studied ^8, 10, 11, 46^. RVs can induce a number of inflammatory mediators that drive Th2 responses in asthma exacerbations. IL-25 is an important mediator in RV-induced asthma exacerbations ^47, 48^. Primary BECs from patients with asthma have increased expression of IL-25, which correlates with the donor atopic status. In mice, blocking the IL-25R following RV infection reduces mucus secretion, airway hyper-responsiveness, and secretion of Th2 cytokines ^8^. The role for IL-33 in driving RV-induced asthma exacerbations was examined in a human experimental model of RV ^10^. RV infection correlated with the inductions of Th2 cytokines and IL-33. Furthermore, infection of primary BECs with RV induced IL-33 secretion. Supernatant transfer from the infected BECs to human T cells or ILC2s strongly induced Th2 responses ^10^. The importance of IL-33 in driving Th2 immune responses is supported by mouse models with deficiencies in the IL-33 pathways ^9^. Human lung epithelial cells infected with RV secrete IL-33 and TSLP ^11^. Furthermore, mice that are simultaneously exposed to ovalbumin and RV show a reduction in regulatory T-cell activation. This reduction is associated with increased Th2 responses and a prevention of ovalbumin tolerance that are driven by IL-33 and TSLP ^11, 27^. In humans with uncontrolled asthma, the inhibition of Th2 responses using tezepelumab (monoclonal antibody specific for TSLP) reduces the rates of asthma exacerbations in patients with asthma inadequately treated with long-acting beta-agonists and medium to high doses of inhaled corticosteroids ^49^. Overall, these studies show the pivotal role of cytokines secreted by epithelial cells in driving the type of innate and adaptive immune responses during RV infection.

In the past few decades, neutrophils have been shown to form neutrophil extracellular traps (NETs) in order to trap invading pathogens such as bacteria through the release of dsDNA, anti-microbial proteins, and histones. The regulation of these NETs is driven by neutrophil elastase and myeloperoxidase ^50– 52^. In addition to trapping bacterial pathogens, NETs have an increasingly important role during viral infections ^53^. The presence of NETs after RV infection and the role they have in driving Th2 immune responses were studied in mice and humans ^46^. RV infection induces NET-associated dsDNA in humans. Patients with asthma challenged with RV-16 showed higher levels of lavage dsDNA, which correlated with cold symptom severity, presence of Th2 cytokines, and asthma exacerbation. Using a mouse allergen-induced model, the same authors showed that RV-infected allergic mice have higher levels of lavage dsDNA in the airways ^46, 54^. When these mice were treated with DNase (to remove NETs) or elastase inhibitors (to prevent NET formation), the Th2 immune responses, production of cytokines, and cell recruitment were diminished. Evidence suggests that NET formation can provide a novel therapeutic target by which DNase, elastase inhibitors, and neutrophil trafficking inhibitors can be used during RV-induced asthma exacerbations.

Multiple asthma mouse models improved understanding of the mechanisms involved in driving Th2 responses during RV infection. The role of γδT cells was studied in human and animal models ^55^. Levels of γδT cells are elevated in asthma and asthma mouse models. Blocking γδT cells in mice enhanced Th2 immune cell recruitment to the airways. Overall, the data suggest that γδT cells are negative regulators of disease during RV-induced asthma exacerbations ^55^. To further characterise the importance of T-cell responses, mice deficient in T-Box expressed in T cells (Tbet), a controller of Th1 cells, were studied in an RV infection model. Mice that lacked Tbet developed a Th2/Th17 phenotype after RV infection ^56^. The lack of Th1 responses is associated with increased viral load, eosinophilia, and mucus production. These findings suggest that weakened Th1 responses, with consequent Th2/Th17 responses, may have an important role in driving allergic features during RV asthma exacerbations ^56^. The phenotype of T helper cell responses is critical for the outcome of RV infection. The recruitment of Th1 cells and the relevant production of IFN-γ have been linked to efficient viral clearance. Studies suggest that viral shedding is inversely related to T-cell counts in the airways ^4^. Finally, an interesting feature of T-cell immunity against RV infections is that they can become activated by shared viral epitopes which can allow potent responses across serotypes ^57^.

## Vaccine approaches against rhinovirus

For decades, vaccine development against RVs has been considered almost impossible ^58^. The large breadth of RV serotypes is a major obstacle in developing therapy. Neutralising humoral responses against RV infection are associated with protection; however, the mechanisms of their induction are poorly understood. Upon RV infection, IgG and IgA are observed in the serum and the airways, respectively ^59^. High levels of serotype-specific antibodies are associated with protection. Despite this, the main limiting factor with humoral responses against RV is that, owing to the high number of serotypes, little cross-reactivity is elicited by neutralising antibodies ^60^.

Recent advances in understanding RV serotypes and viral capsid structures provide promising vaccine targets. The amino acid identity within RV serotypes is at 70%, and VP1 and VP4/VP2 (VP0) capsid regions are the most conserved ^61^. A recombinant VP0 vaccine in conjunction with an IFA/CpG adjuvant in mice elicited strong cross-reactive Th1 responses and, following virus challenge, enhanced neutralising antibody responses within the serotype ^62^. The usage of immunogens, such as VP0, in combination with Th1-promoting adjuvants provides a promising avenue for RV vaccine development ^60, 63^. The main limitation with vaccines against RV is increasing the breadth of the immune responses across other serotypes. For example, vaccination of rabbits with VP1/VP3 increases neutralising antibodies only within a specific group of serotypes ^64^. Taken together, these results highlight the difficultly for a single antigen providing protection across all RV serotypes ^65^. Through the use of an adjuvanted polyvalent RV vaccine in macaques and mice, the induction of neutralising antibodies across a diverse range of RV serotypes is feasible ^64^. The identification of conserved regions within the RV genome and production of an adjuvanted polyvalent RV vaccine provide exciting pathways for RV vaccine development. Overall, recent advances in understanding RV immunity increase hopes that a vaccine may be feasible after all.
